# Restricted kinematic alignment in total knee arthroplasty achieves equivalent or superior functional outcomes to mechanical alignment without compromising implant survivorship: A systematic review

**DOI:** 10.1016/j.jor.2026.02.025

**Published:** 2026-02-04

**Authors:** Amaan A. Merchant, Maryam Imran, Mihika Joshi, Padmanabhan Subramanian, Farhad Iranpour

**Affiliations:** a University College London Medical School, London, United Kingdom; b Royal Free London NHS Foundation Trust, Trauma & Orthopaedics, London, United Kingdom

**Keywords:** Restricted kinematic alignment, Mechanical alignment, Total knee arthroplasty, Outcome, Survivorship

## Abstract

**Purpose:**

This systematic review assesses the viability of restricted kinematic alignment compared to mechanical alignment by assessing clinical outcomes, radiological findings, and patient-reported outcome measures. Median follow-up was 20.3 months. Both RCTs and observational studies are included in this review.

**Methods:**

A search was carried out according to PRISMA guidelines over PubMed, Embase, Cochrane and Web of Science databases. The following search string, (‘restricted kinematic alignment’ AND ‘mechanical alignment’) AND (‘total knee arthroplasty’ OR ‘total knee replacement’) was used. Titles, abstracts and keywords were screened against predetermined inclusion and exclusion criteria. Data was extracted by two independent reviewers and conflict was resolved by consensus.

**Results:**

A total of 110 papers between 1975 and January 2025 were screened with 11 texts included in the final analysis.

Restricted kinematic alignment achieved superior FJS (p = 0.044) and KSS (p = 0.028) at 12 and 24 months respectively compared to mechanical alignment. Radiographic analyses confirmed coronal limb alignment and femoral/tibial component positioning remained within defined safe zones, with no significant increase in outliers. Importantly, complication and revision rates did not differ significantly between groups, indicating no compromise in short-term safety which is defined as follow-up less than five years.

**Conclusion:**

This analysis suggests restricted kinematic alignment provides functional advantages whilst adhering to strict ‘intraoperative safe zones’ with no increase in short-term complications. However, surgical technique remains variable with some studies using robotic assisted surgery and others utilising calipered kinematic adjustments or navigation-assisted resections. Long-term implant durability also remains a crucial unanswered question.

**Level of evidence:**

Level III

## List of abbreviations in the text

E-rKA: Extended Restricted Kinematic Alignment
FJS: Forgotten Joint Score
FA: Functional Alignment
GB: Gap Balancing
HKA: Hip-knee-ankle Angle
irKA: Inverse Restricted Kinematic Alignment
JLOA: Joint Line Orientation Angle
KA: Kinematic Alignment
KOOS: Knee Injury and Osteoarthritis Outcome Score
KSS: Knee Society Score
LDFA: Lateral Distal Femoral Angle
LoE: Levels of Evidence
MA: Mechanical Alignment
MPTA: Medial Proximal Tibial Angle
NOS: Newscastle-Ottowa Scale
OKS: Oxford Knee Score
PC: Posterior Condyles
PTS: Posterior Tibial Slope
PROMs: Patient Reported Outcome Measures
PRISMA: Preferred Reporting Items for Systematic Reviews and Meta-Analyses
RCT: Randomised Controlled Trial
rKA: Restricted Kinematic Alignment
RoB2: Cochrane Collaborative Risk of Bias 2
TA: Trochlear Angle
TEA: Transepicondylar Axis
TKA: Total Knee Arthroplasty
VAS: Visual Analog Score
WOMAC: Western Ontario and McMaster Universities Osteoarthritis Index

## 1 Introduction

Mechanical alignment (MA) in TKA aims for a neutral hip–knee–ankle (HKA) axis (180°) to evenly distribute load and maximise implant longevity.^1^ Despite long-term survival (>90% at 15 years),^2^ up to ∼20% of patients remain dissatisfied, possibly due to altered knee kinematics from forcing native anatomy into a “one-size-fits-all” alignment.^3^ Recent studies have challenged the traditional assumptions of MA, highlighting the variability in patients' preoperative knee anatomy, and suggest that strict neutral alignment may not be essential for successful long-term outcomes 4, 5, 6. Especially considering that neutral alignment is naturally occurring in only about 5–6% of knees. This has prompted exploration of alternatives that accommodate individual anatomical variations.^7^^,^^8^ Kinematic alignment (KA), introduced by Howell et al.^9^ attempts to restore each patient's unique pre-arthritic joint line and axes of rotation. However, unrestricted KA risks extreme residual varus/valgus in some knees, raising concerns about implant loading. To address this, restricted kinematic alignment (rKA) has been proposed primarily by Vendittoli et al.,^10^ preserving native kinematics within ±3° of neutral coronal alignment. KA has demonstrated excellent long-term implant survivability, with only a 7% reoperation rate at 16 years follow-up.^11^ It is yet to be seen whether rKA can provide equivocal or improved survivorship (see Table 1).

**Table 1:** Overview of the design characteristics of included articles.^a^.

Author (year)	Study Design (LOE)	Intervention	Single surgeon/team	Follow up (months)	N TKA's	N MA	N rKA	Implant type	Guide type	rKA algorithm
T. Kobayashi (2024)^9^	RS (IV)	Surgical	Yes	36	114	49	65	GMK Sphere, PS medial pivot (Medacata).	CT-based PSG	Venditolli's algorithm. In cases where the planned coronal resection angle was outside the rKA target zone, femoral anatomy prioritised over tibial alignment
T. Matsumoto (2020)^10^	RCT (I)	Surgical	NR	12	60	30	30	e-motion, CR (B. Braun Aesculap)	Computer assisted navigation	3-degree varus and 7-degree posterior slope in tibial cut
S. Abhari (2021)^11^	RS (III)	Surgical	Yes	17 (12-27)	230	115	115	Triathlon, CR (Stryker)	Robotic assisted	Vendittoli's algorithm
SJ. MacDessi (2020)^12^	RCT (I)	Surgical	No	12	138	68	70	Legion, PS (S&N)	Computer assisted navigation	Safe zones defined as: LDFA: 4° valgus to 3° varus MPTA: 3° valgus to 4° varus HKA: 5° varus to 4° valgus
R. Ma (2022)^13^	RS (III)	Surgical	Yes	6	93	45	48	Legion, CR (S&N)	Computer assisted navigation	Vendittoli's algorithm
M. Ettinger (2024)^14^	RCT (II)	Surgical	Yes	24	98	51	47	GMK Sphere, medial pivot (Medacata)	CT-based PSG	Vendittoli's algorithm
E. Sappey-Marinier (2021)^15^	RS (III)	Surgical	Yes	53 (MA) vs 43 (KA)	150	100	50	GMK, PS (Medacta)	CI; PSG	Vendittoli's algorithm
PW. De Grave (2020)^16^	RS (III)	Surgical	No	12	80	40	40	Triathlon, CR (Stryker)	Robotic assisted	irKA approach. Maintain HKA angle within 174° to 183°. Restore MPTA within 84° to 92
I. Shichman (2024)^17^	RS (III)	Surgical	No	>12	200	100	100	ATTUNE, PS (Depuy-Synthes)	rKa: Robotic assisted vs. MA: CI	irKA approach
A. Parente (2023)^18^	RS (III)	Surgical	No	24	120	80	40	Persona, PS (Zimmer-Biomet) No patellar resurfacing	CI	irKA approach
Treu (2023)^19^	RS (III)	Surgical	Yes	NA	1925	1223	702	NR	Computer assisted navigation	Femoral target alignment: medial compartment OA - goal of 2° varus, lateral compartment OA - goal of 2° valgus

a N, number; RS, retrospective study; RCT, randomised control trial; CS, cross-sectional; PS, prospective study; NA, not applicable; NR, not reported; MA, mechanical alignment; rKA, restricted kinematic alignment; PS, posterior stabilised; CR, cruciate retaining; PSG, patient-specific guide; CI, conventional instrument; LDFA, lateral distal femoral angle; MPTA, medial proximal tibial angle; HKA, hip-knee-ankle; irKA, inverse restricted kinematic alignment; OA, osteoarthritis.

Recent evidence on rKA has been synthesised in several reviews. Risitano et al.^12^ analysed 475 knees and concluded that rKA provides “equivalent or slightly better” PROMs than MA without increased short-to mid-term failure. Similarly, Cortina et al.^13^ included 892 knees and found postoperative PROMs were “not inferior or even better” for rKA, with higher Forgotten Joint Scores and satisfaction with no increase in complications. Thus, current evidence suggests rKA may modestly improve function without compromising safety. However, gaps remain. A recent umbrella review of kinematic alignment strategies noted that “the current literature is inadequate to determine” any clear advantage of KA philosophies over MA, citing methodological flaws in existing analyses.^14^

Purpose: This systematic review aims to determine whether rKA can enhance PROMs and patient satisfaction without increasing malalignment or revision risk over short-to mid-term follow-up, whilst incorporating the most recent comparative trials to assess whether these findings reinforce the existing body of literature.

## 2 Materials and methods

In keeping with the Preferred Reporting Items for Systematic review and Meta-analysis (PRISMA) guidelines, a literature search was conducted on PubMed, Embase, Cochrane and Web of Science databases for publications between 1975 and 2025 using the following search string; (‘restricted kinematic alignment’ AND ‘mechanical alignment’) AND (‘total knee arthroplasty’ OR ‘total knee replacement’). Two independent reviewers XXBLINDEDXX screened for eligibility at each stage with conflicts resolved through consensus.

**Fig. 1:**
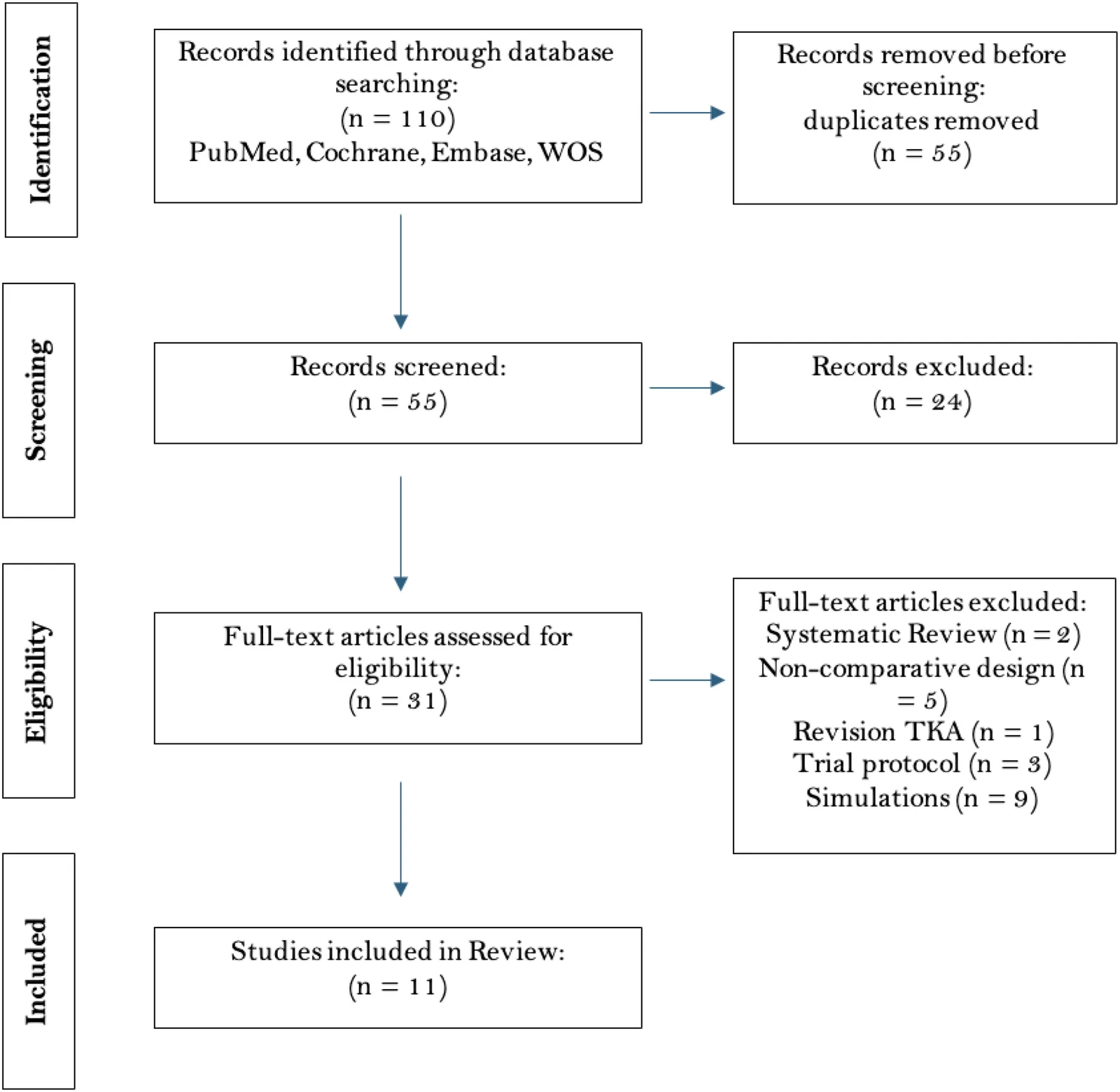
Flow chart of search and screening process.

The search yielded 110 papers. Rayyan (Rayyan Systems Inc) screening software was used for handling abstracts and papers during the screening process. After duplicates were removed, the full texts of 31 included studies were assessed for inclusion or exclusion against predetermined criteria. Eligibility criteria included full text articles published in English that reported clinical and/or radiological outcomes comparing rKA and MA. Non-comparative studies and revision arthroplasty studies were excluded. RCTs were prioritised, with select observational studies also included. Observational studies included were comparative in nature and reported clinical and/or functional outcomes. Case reports, letters, systematic reviews, meta-analyses and conference abstracts were all excluded. 11 clinical studies were included in the systematic review ^8^^,^^15^, ^16^, ^17^, ^18^, ^19^, ^20^, ^21^, ^22^, ^23^, ^24^. This process is summarised in Fig. 1.

Data was extracted from full text manuscripts by two independent reviewers XXBLINDEDXX. The clinical scores extracted were WOMAC, OKS, KSS, FJS, EQ-5D, KOOS and VAS. The primary endpoint was implant survivorship and secondary endpoints were infection, aseptic loosening, implant wear and instability. Each included study was also analysed according to the Levels of Evidence (LoE) of the Oxford Centre for Evidence-Based Medicine 2011.^25^ Risk of bias of included studies was assessed by two reviewers using the Cochrane Collaborative Risk of Bias 2 (RoB2) and Newcastle-Ottawa Scale (NOS) tools for randomised controlled trials (RCTs) and observational cohort studies respectively.^26^^,^^27^ Extracted data was analysed using pooled analysis of mean differences for PROMs. A protocol was published retrospectively.

## 3 Results

### 3.1 Demographic data

A total of 3208 knees were included, of which 1901 received MA and 1307 received rKA. The average age of patients receiving MA was 69.2 years (range, 63 - 75.5) whilst the average age of patients receiving rKA was 70.4 years (range, 67 - 76.4). The MA population had an average BMI of 29.8 kg/m^2^ (range, 25.2 - 35) with the average BMI being 29.2 kg/m^2^ (range, 26.4 - 32) in the rKA population. A male: female ratio of 999:835 and 594:644 for the MA and rKA groups respectively was observed across studies which reported on these domains. The average follow-up was 20.3 months (range, 6-53).

Few studies report or analyse postoperative outcomes in the context of demographic data. Winnock De Grave et al. reported decreasing postoperative Oxford Knee Score (OKS) with univariable analysis when adjusted for age (p = 0.047) and worse OKS for women on multivariable analysis (p = 0.045).^22^ Ma et al. also carried out demographic subgroup analysis and found no significant difference for gender and BMI on Knee Injury and Osteoarthritis Outcome Score (KOOS), EQ (5D) and Forgotten Join Score (FJS).^19^ However, their analysis did reveal worse outcomes on these aforementioned PROMs when adjusted for age (p < 0.05).^19^ This is, however, insufficient evidence to ascertain whether age, gender or BMI have an effect on PROMs following TKA. Furthermore, there was no analysis within the included studies in relation to radiological outcomes. Other studies have supported the role of age, gender and BMI in increasing the rate of adverse outcomes following TKA, but this needs further consideration.^28^

### 3.2 Bias assessment of included studies

**Fig. 2:**
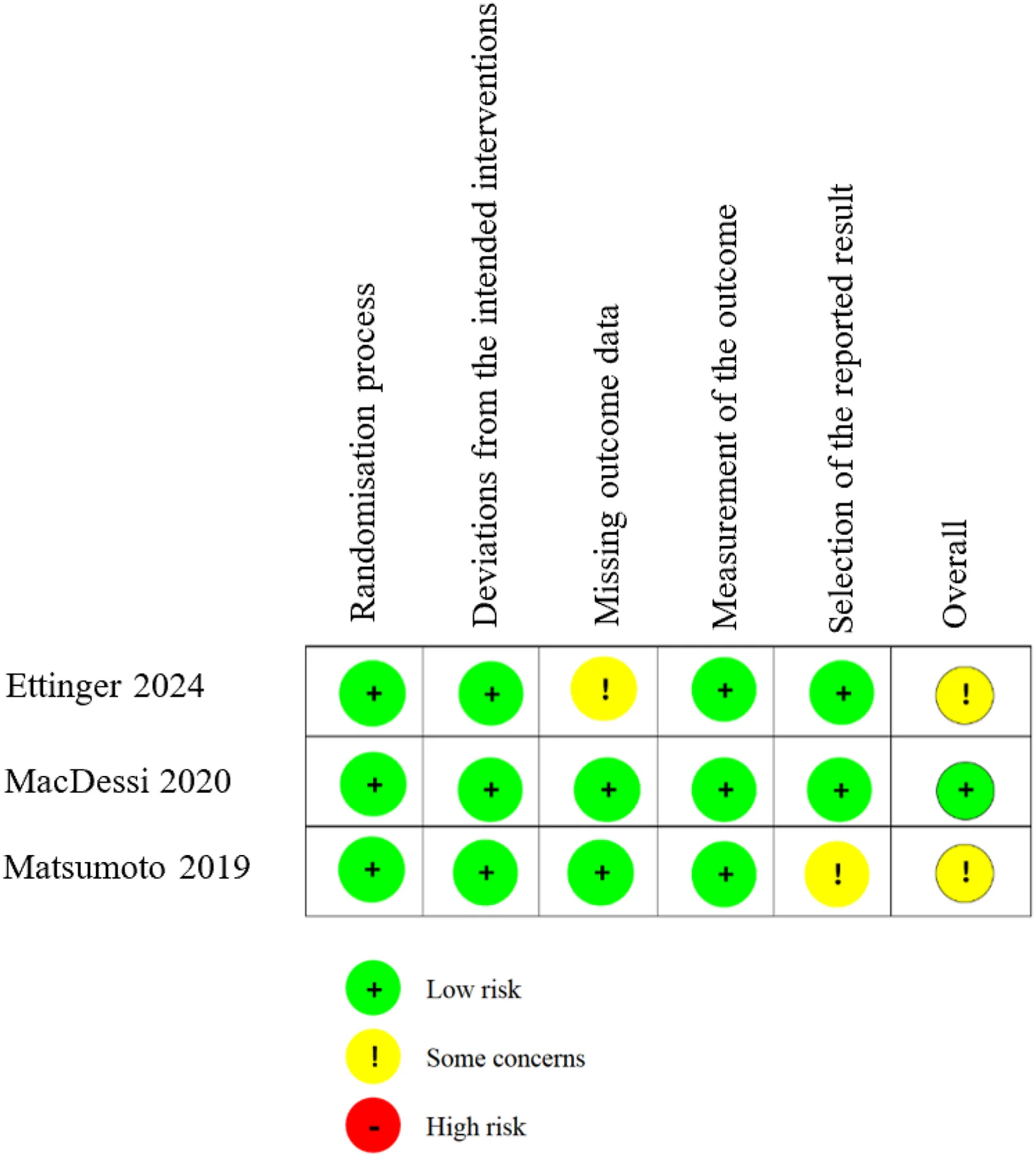
Risk of Bias 2 summary of RCTs.

**Fig. 3:**
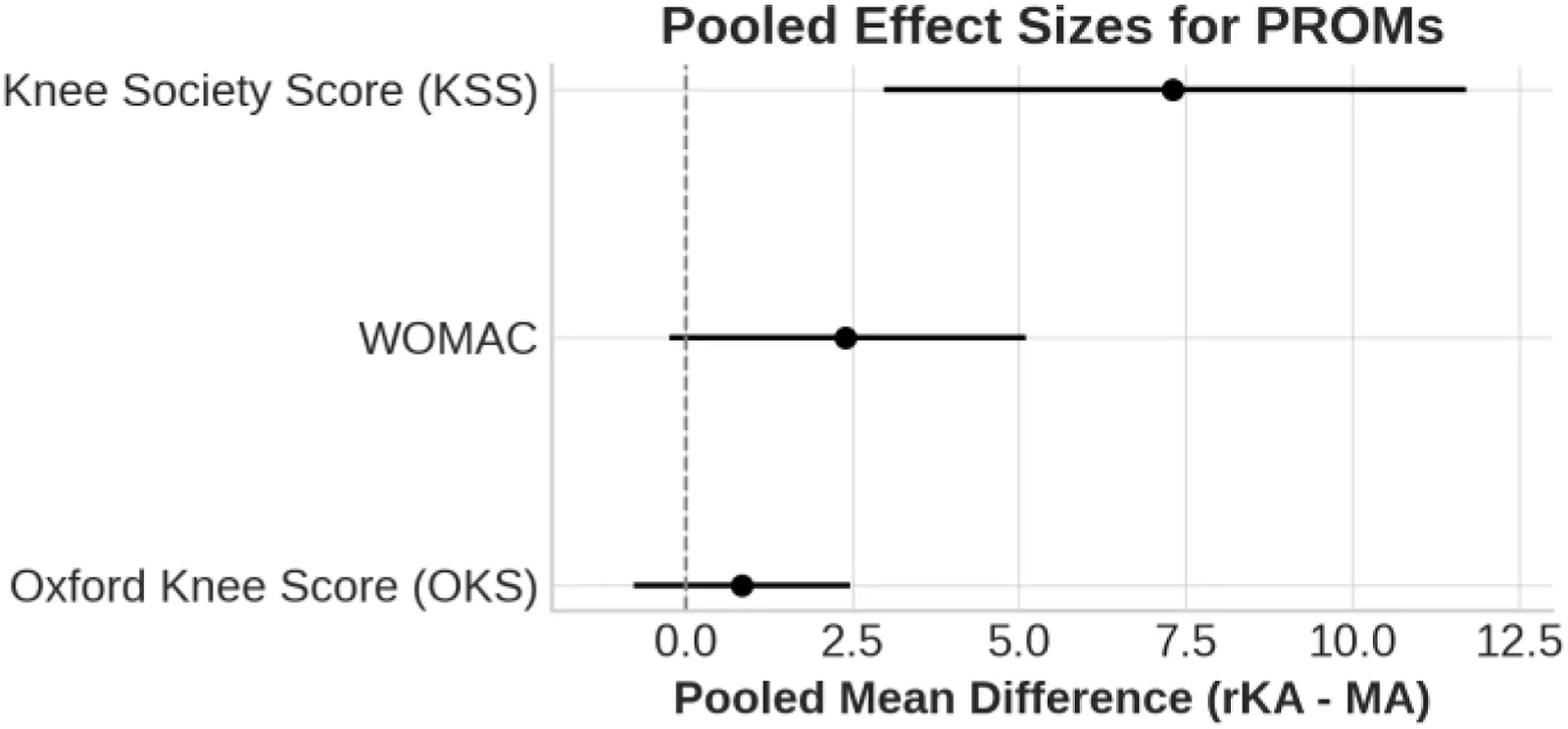
Pooled mean difference of PROMs.

Of the three included RCTs, one had a low risk of bias as assessed with RoB-2 and two had some concerns as seen in Fig. 2. Due to the nature of the intervention, surgeons were not blinded at the point of delivery. This was mitigated by Matsumoto et al.^16^ who reported clear blinding of intraoperative outcome assessors. Ettinger et al.^20^ had a relatively small sample size with a loss to follow-up of more than 5% (see Fig. 3).

The NOS tool includes assessment of three aspects: selection of study subjects, comparability between groups and outcome measurement. The total score is 9 with 5 – 9 accepted as higher quality observational research. According to the NOS criteria, all 17 included cohort studies had a score ≥5 which is considered to be the acceptable standard to minimise risk of bias.

### 3.3 Clinical scores

Five studies reported KSS outcomes, of which three demonstrated results favouring rKA. Abhari et al.^17^ observed significant improvements in both clinical and functional KSS subscales in the rKA group. Specifically, the clinical KSS improved from 41 ± 9.4 to 93 ± 9.4, and the functional KSS improved from 51 ± 10 to 85 ± 16, both with p-values <0.001. Matsumoto et al.^16^ also reported significantly higher functional scores in rKA patients (73.6 ± 13.7) compared to those who received MA (63.8 ± 18.8, p = 0.04), alongside significantly better outcomes in objective indicators (p < 0.001), patient satisfaction (p = 0.036), and overall functional activity (p = 0.008). Ettinger et al.^20^ demonstrated statistically significant improvements in the satisfaction and expectation subscales at both 12 and 24 months in the rKA group, with KSS function showing significant improvement at 24 months (84.3 ± 12.5 for rKA vs. 77.9 ± 13.4 for MA, p = 0.028). The same study reported satisfaction scores of 34.0 ± 6.7 (rKA) vs. 30.7 ± 7.1 (MA) and expectation scores of 10.4 ± 2.7 (rKA) vs. 9.1 ± 2.5 (MA), both favouring rKA with p-values <0.05.

Two studies reported no significant differences in KSS between the alignment strategies. Sappey-Marinier et al.^21^ observed improvements in both groups (52.0 ± 30.4 vs. 57.5 ± 29.8), but without statistically significant differences across any of the subscales. Similarly, Parente et al.^24^ reported no significant difference in subjective KSS (89.9 ± 2.6 for rKA vs. 88.5 ± 4.6 for MA) or functional KSS (88 ± 4.1 vs. 86.6 ± 8.5).

Pooled analysis from the RCTs yielded a significant mean difference in KSS favouring rKA, with a pooled mean difference of 7.3 points (95% CI: 2.97 to 11.70).

Two studies assessed outcomes using the WOMAC score. Abhari et al.^17^ reported a substantial improvement in WOMAC scores for the rKA group, increasing from 43 ± 15 to 90 ± 11 postoperatively, compared with a postoperative score of 85 ± 1.8 in the MA group (p = 0.039). In contrast, Ettinger et al.^20^ found no significant differences at either 12 or 24 months postoperatively. The pooled analysis demonstrated a non-significant trend favouring rKA, with a mean difference of 2.4 points (95% CI: −0.24 to 5.10).

KOOS outcomes were reported in five studies. Abhari et al.^17^ found a significant improvement in KOOS for the rKA group compared to the MA group (87 ± 15 vs. 78 ± 1.9, p = 0.001). Ma et al..^19^ reported a statistically significant improvement at both early (T1, 10 days post-op) and later (T6, 6 months post-op) follow-up points. At T1, KOOS improved by 26.053 in the rKA group and 18.607 in the MA group (p < 0.001), and at T6, the improvements were 51.017 and 46.896 respectively (p = 0.023). However, three studies—Kobayashi et al.,^15^ Parente et al.,^24^ and MacDessi et al.^18^ - found no significant differences in KOOS scores between the two alignment groups.

Four studies assessed the Forgotten Joint Score (FJS). Ettinger et al.^20^ reported a significant difference favouring rKA at 12 months post-op (62.2 ± 22.9 vs. 52.4 ± 23.8, p = 0.044), though this difference was not sustained at 24 months (65.1 ± 23.2 vs. 56.9 ± 26.9, p = 0.127). The remaining studies—Kobayashi et al.,^15^ Ma et al.,^19^ and MacDessi et al.^18^ - did not demonstrate any statistically significant differences in FJS scores between rKA and MA.

Oxford Knee Score (OKS) was assessed in three studies,^15^^,^^20^^,^^22^ none of which found a statistically significant difference between the two alignment methods. EQ-5D scores were reported in two studies.^18^^,^^19^ Ma et al.^19^ found significantly greater improvements in the rKA group at both 10 days (0.457 vs. 0.367, p < 0.001) and 6 months (0.606 vs. 0.565, p = 0.01) postoperatively, whereas MacDessi et al.^18^ observed no difference. Visual Analogue Scores (VAS) were reported in two studies. Winnock De Grave et al.^22^ reported significantly better pain scores in the rKA group (9.2 ± 0.8 vs. 8.5 ± 1.3, p = 0.012) using a tibia-first technique (inverse restricted kinematic alignment). Parente et al.^24^ found no statistically significant difference (see Table 2).

### 3.4 Lower limb alignment

Several radiographic measurements were recorded for analysis of lower limb alignment, as seen in Table 3. These included HKA, lateral distal femoral angle (LDFA) and medial proximal tibial angle (MPTA). Amongst rKA participants, the average preoperative and postoperative HKA was 174.0 and 178.5, respectively. The average preoperative and postoperative LDFA was 88.5 and 89.4, respectively. The average preoperative and postoperative MPTA was 86.7 and 88.1, respectively. In the MA group, the average preoperative and postoperative HKA was 174.6 and 179.0, respectively. The average preoperative and postoperative LDFA was 88.7 and 89.8, respectively. The average preoperative and postoperative MPTA was 86.8 and 89.7, respectively.

**Table 2:** Overview of reported PROMs results.^a^.

Author (year)	Objective tests and/or joint line parameters	Complications and/or implant survival in favour of	PROMs in favour of						
			WOMAC	OKS	KSS	FJS	EQ-5D	KOOS	VAS
T. Kobayashi (2024)^9^		Neither		N		N		N	
T. Matsumoto (2020)^10^	Significant improvement in postoperative flexion angle with rKA				rKA				
S. Abhari (2021)^11^			rKA		rKA			rKA	
SJ. MacDessi (2020)^12^	Improved quantitative knee balance with rKA					N	N	N	
R. Ma (2022)^13^		Neither				N	rKA	rKA	
M. Ettinger (2024)^14^		Neither	N	N	rKA	rKA			
E. Sappey-Marinier (2021)^15^		Increased risk of aseptic tibial loosening with rKA. Overall implant survivorship was significantly higher with MA			N				
PW. De Grave (2020)^16^	PASS thresholds achieved by greater proportion with rKA	Neither		N					rKA
A. Parente (2023)^18^		Neither			N			N	N

a PROMs, patient reported outcome measures; PASS, patient acceptable symptom state; JLO, joint line obliquity; JLH, joint line height; MA, mechanical alignment; rKA, restricted kinematic alignment; N, neither; WOMAC, Western Ontario and McMaster Universities Osteoarthritis Index; OKS, Oxford Knee Score; KSS, Knee Society Score; FJS, Forgotten Joint Score; EQ-5D, EuroQol- 5 Dimension; KOOS, Knee Injury and Osteoarthritis Outcome Score; VAS, Visual Analog Score.

**Table 3:** Overview of reported radiographic/alignment results.^a^.

	HKA				LDFA				MPTA				Additional Measurements
	MA		rKA		MA		rKA		MA		rKA		
Author (year)	pre (SD)	post (SD)	pre (SD)	post (SD)	pre (SD)	post (SD)	pre (SD)	post (SD)	pre (SD)	post (SD)	pre (SD)	post (SD)	
T. Kobayashi (2024)^9^	171.3 (5.4)	179.4 (2.6)	170.6 (5.1)	177.9 (1.7)	91.8 (2.6)	90.0 (2.2)	91.2 (2.5)	90.5 (3.1)	83.7 (2.8)	90.0 (1.8)	84.2 (2.6)	86.9 (2.2)	JLOA: MA: −0.8 (2.8) pre & −4.1 (1.6) post vs. rKA: −0.3 (2.7) pre & −2.0 (2.0) post
T. Matsumoto (2020)^10^	170.1	179.4 (2.4)	169.7	178.1 (2.0)	89.6	89.8 (1.8)	89.5	91.8 (1.1)	87.1	89.9 (1.3)	87	86.7 (1.1)	Tibial internal rotation: MA: 15.8 (10.1) vs. rKA: 21.1 (7.5)
S. Abhari (2021)^11^	NR		173	177	NR								Tibial Alignment: rKA: 2.0 (pre) & 2.0 (post)
SJ. MacDessi (2020)^12^	176.3	179.4 (2.3)	177.2	179.8 (2.3)	87.6	90.6 (1.5)	87.5	89.2 (1.8)	87.3	90.0 (1.9)	87.8	88.9 (1.8)	ICPD (PSI): at 10° flexion: MA 32.0 & rKA 11.7 vs. at 45° flexion: MA 25.2 & rKA 14.8 vs. at 90° flexion: MA 19.1 & rKA 11.7
R. Ma (2022)^13^	172.2 (8.0)	178.0 (4.0)	171.1 (6.6)	179.2 (2.7)	89.7 (5.5)	91.5 (3.3)	90.1 (3.3)	90.5 (1.8)	85.0 (3.1)	89.4 (2.5)	85.0 (2.8)	89.7 (1.9)	Surgical accuracy for rKA: 0.5° (95% CI: 0.4–0.7) Surgical accuracy for MA: 3.5° (95% CI: 2.7–4.3)
M. Ettinger (2024)^14^	175.7 (3.3)	178.1 (1.7)	174.4 (3.1)	177.7 (2.2)	87.4 (2.0)	90.3 (1.7)	85.6 (13.3)	87.5 (2.2)	86.8 (6.0)	88.8 (1.7)	86.2 (1.6)	86.5 (2.4)	
E. Sappey-Marinier (2021)^15^		179.3 (2.7)		178.8 (3.8)		89.8 (1.3)		91.3 (2.4)		89.5 (0.9)		88.6 (2.6)	Tibial Slope: MA post: 88.6 (1.1) & rKA post: 86.6 (1.4) Patellar Tilt: MA post: 0.7 (3.5) & rKA post: 0.8 (3.4)
PW. De Grave (2020)^16^	176.9 (4.6)	179.6 (1.9)	176.3 (4.3)	178.3 (2.1)	87.7 (1.4)	90.0 (1.6)	88.0 (1.4)	88.8 (1.4)	87.4 (1.7)	89.6 (0.9)	86.7 (1.3)	87.1 (1.4)	Femoral Rotation from PCA: aMA: post: 4.8° (2.3) & rKA: post: 2.3° (1.4) Tibial Slope: aMA: 4.1° (1.6) & rKA: 4.2° (1.2) Medial Tibial Resection: aMA: 4.4 mm (1.2) & rKA: 5.4 mm (0.9) Lateral Tibial Resection: aMA: 6.2 mm (1.3) & rKA: 6.1 mm (1.2)
I. Shichman (2024)^17^	NR												MA: JLO: pre: 2.94 (1.9) & post: 2.31 (1.5) JLH: pre: 40.6 mm (4.5) & post 40.6 mm (4.8) rKA: JLO: pre: 2.43° (1.8) & post 2.30° (1.4) JLH: pre: 41.2 mm (6.6) & post: 42.4 mm (6.2)
A. Parente (2023)^18^	179.5 (2.5)	179.0 (2.0)	180.0 (3.0)	179.5 (2.5)	87.0 (1.5)	86.5 (1.0)	87.5 (2.0)	87.0 (1.5)	90.5 (1.5)	90.0 (2.0)	90.0 (2.0)	90.5 (1.5)	Insall Index: irKA: pre: 1.0 (0.2) & post 1.1 (0.1) vs. aMA: pre: 1.1 (0.2) & post 1.1 (0.2) Caton-Deschamps (C-D) Index: irKA: pre: 0.8 (0.2) & post: 1.0 (0.1) vs. aMA: pre: 0.9 (0.2) & post: 1.0 (0.2)
Treu (2023)^19^	NR												ABN (rKA) cohort and CONV (MA) cohort: neutral, valgus and varus rates

a All values given in degrees, unless specified. HKA, Hip Knee Ankle Angle; LDFA, Lateral Distal Femoral Angle; MPTA, Medial Proximal Tibial Angle; MA, Mechanical Alignment; rKA, Restricted Kinematic Alignment; irKA, Inverse Restricted Kinematic Alignment; pre, Preoperative; post, Postoperative; SD, Standard Deviation; NR, Not Reported; JLOA, Joint Line Obliquity Angle; ICPD, Intracompartmental Pressure Difference; PSI, pounds/square inch; CI, Confidence Interval; PCA, Posterior Condyle Angle; JLH, Joint Line Height; TEA. Trans-Epicondylar Axis; E-rKA, Extended Restricted Kinematic Alignment; PTS, Posterior Tibial Slope; FCF, Femoral Component Flexion; FCR, Femoral Component Rotation; TA, Trohlear Angle; ABN, Accelerometer Based Navigation (rKA); CONV, Conventional (MA).

Various other alignment parameters were recorded across studies. Kobayashi et al.^15^ reported on Joint Line Orientation Angle (JLOA) and found a significant difference in postoperative values between the two comparators (in favour of rKA). In the MA group, the mean preoperative JLOA was −0.8° (range: −8.5 to 4.0, SD: 2.8), as compared to −0.3° (range: −6.5 to 4.0, SD: 2.7) in the rKA group (p = 0.326). In the MA group, the mean postoperative JLOA was −4.1° (range: −7.0 to −0.5, SD: 1.6) versus a mean postoperative JLOA of −2.0° (range: −6.0 to 3.0, SD: 2.0) in the rKA group (p < 0.001).

Matsumoto et al.^16^ found a significant improvement in tibial internal rotation between 60 and 120° of flexion in the kinematic group, with a mean rKA value of 21.1° ± 7.5 and a mean MA value of 15.8° ± 10.1 (p = 0.030). Sappey-Marinier et al.^21^ observed a statistical difference in tibial slope between rKA and MA groups (88.6 ± 1.1° in MA versus 86.6 ± 1.4° in rKA, p < 0.001), however found no significant difference in patellar tilt between the two groups (0.7 ± 3.5° in MA versus 0.8 ± 3.4° in rKA).Winnock De Grave et al.^22^ found no significant difference in tibial slope, with a mean MA value of 4.1 ± 1.6° and a mean irKA value of 4.2 ± 1.2°.

### 3.5 Complications

Complication rates were not invariably reported. Sappey-Marinier et al.^21^ reported a 16% revision rate for aseptic tibial loosening amongst rKA participants. All revisions occurred between 11.9 and 25.5 months postoperatively. In the MA group, 2 patients were revised due to aseptic tibial loosening at 38.4 and 45.1 months postoperatively. Additionally, 1 patient developed an early infection 2 months postoperatively, leading to a complete revision with component exchange. 1 patient underwent arthroscopic arthrolysis at 4 months postoperatively due to stiffness. At 50 months follow-up, the overall implant survivorship was significantly higher for the MA group compared to the rKA group (97% vs, 84% respectively, P = 0.002). Kobayashi et al.,^15^ Ma et al.,^19^ Ettinger et al.,^20^ Winnock De Grave et al.^22^ and Parente et al.^24^ reported no significant tendency for complications and/or implant survival between groups.

## 4 Limitations

The following limitations of this analysis were identified. There is disparity in the surgical method used between included studies. For example, robotic-assisted versus manual techniques for rKA have differing algorithms which introduce variability that may influence radiographic alignment/functional outcomes. However, this represents the evolving landscape of technological advancement within surgery. Second, follow-up durations were variable, and there was limited long-term data beyond 5 years, making it difficult to draw definitive conclusions on mid-to long-term implant longevity. Third, the absence of a standardised procedure between studies with differing rKA algorithms used may have influenced pooled analyses. Most studies employed Vendittolli's algorithm ^15^^,^^17^^,^^19^, ^20^, ^21^, whilst others used a tibia-first approach 22, 23, 24. Fourth, two included RCTs had some concerns regarding risk of bias. The relatively small sample size observed in certain studies is clinically significant as it limits statistical power to detect true differences between rKA and MA, particularly for low-incidence outcomes such as revision rates or implant survival. Further, with retrospective analysis there exists inherent risk of selection bias, confounding, and limited control over variables, reducing the strength of causal inferences. Lastly, while this review provided a comprehensive comparison of rKA and MA, future RCTs with standardised protocols are needed to further delineate optimal surgical strategies.

## 5 Discussion

This systematic review critically evaluates whether rKA provides both biomechanical and clinical advantages over MA whilst mitigating the complications associated with unrestricted KA. rKA demonstrates functional outcomes that are equivalent or superior to MA across key PROMs, particularly in KSS and early joint awareness measures such as the Forgotten Joint Score. Radiographic analyses confirm that rKA maintains alignment within safe coronal and sagittal parameters, alleviating long-standing concerns about component malposition or excessive varus deviation. Importantly, no increase in complication or revision rates was observed, with mid-to long-term implant survivorship exceeding 98% in large prospective cohorts 31, 32. These findings suggest that rKA may offer meaningful patient-centred benefits without compromising mechanical safety or implant longevity, provided surgical execution is precise and patient selection is appropriate.

### 5.1 PROMs and clinical outcomes

Multiple studies demonstrated improved functionality, early proprioception and joint awareness in favour of rKA.^16^^,^^17^^,^^20^ PROMs highlighted this consistently, with rKA either equivocal or superior to MA across various domains.^16^^,^^17^^,^^19^^,^^20^^,^^22^ For example, rKA has shown statistically superior KSS function scores at 24 months (p = 0.028),^20^ higher FJS at 12 months (indicating improved neurophysiological adaptation to the prosthesis)^20^ and non-inferiority across WOMAC, KOOS and UCLA activity scores, suggesting that rKA does not compromise functional recovery. However, a key surgical consideration is whether these apparent improvements translate into long-term biomechanical advantages or whether they are transient benefits attributable to early adaptation and soft tissue accommodation.

### 5.2 Radiographic outcomes

One of the fundamental concerns associated with rKA is the potential for excessive coronal deviation, particularly in the varus knee phenotype. Results demonstrated, however, that postoperative HKA remains within ±3° of neutral across most studies, supporting rKA's claim of maintaining a controlled alignment philosophy ^15^^,^^17^^,^^19^, ^20^, ^21^. MPTA and LDFA adjustments ensured that resections remained within implant tolerances, mitigating concerns over component malpositioning. Nonetheless, an important surgical nuance emerged in the strategy employed for tibial resection. Unlike MA, which relies on perpendicular tibial cuts, rKA retains a slight native varus in constitutional varus knees, potentially altering load distribution across the polyethylene insert. This then raises concerns over asymmetrical wear patterns, especially in cases where posterior tibial slope (PTS) and component rotation are not precisely optimised.^29^^,^^30^ Whilst short-term radiographic and alignment assessments suggested this approach remained within safe tolerances, long-term polyethylene wear studies will be imperative in determining whether this strategy has an impact on implant survivorship.

### 5.3 Surgical implications

Whilst rKA seemingly provided tangible functional benefits, its intraoperative execution was highly variable. A critical distinction across studies was the lack of a universal rKA technique. Some surgeons utilised robotic-assisted planning whereas others employed calipered kinematic adjustments or navigation-assisted resections 16, 17, 18, 19^,^^22^^,^^23^. This raised several key surgical considerations; whether rKA should follow a systematic algorithm for tibial slope and coronal cut restrictions or be case-dependent, whether rKA should strictly adhere to native posterior condylar reference points or if adjustments should be made based on intraoperative soft tissue tensioning, and whether rKA should be used preferentially in cases with preoperative ligament imbalance (i.e. medial laxity in varus knees), given that rKA theoretically reduces the requirement for soft tissue releases.^10^ Addressing these questions is vital, as inconsistent surgical execution may introduce variability in clinical outcomes which will increase the difficulty of establishing rKA as a new standard.

### 5.4 Complications and implant longevity

One of the most controversial aspects of rKA identified, was its implications for implant longevity. Results suggested no statistically significant difference in complication rates between rKA and MA however isolated studies raised concerns about aseptic tibial loosening ^15^^,^^19^, ^20^, ^21^, ^22^^,^^24^. The biomechanical rationale behind this concern is that retaining slight native varus alignment may increase compressive forces on the medial tibial plateau, potentially exceeding polyethylene or cement tolerances over time. Despite this, cases of implant loosening have not been consistently observed across studies, suggesting that patient selection, component, design and surgical technique may have been confounding variables. Notably, no included study reported an exceedingly high failure rate with rKA, nor was there a clear signal for increased revision risk. Morcos et al.^31^ conducted a prospective, longitudinal cohort investigation of 104 consecutive primary rKA TKAs. Computer-assisted navigation (Orthomap ASM, Stryker) and cemented cruciate-retaining implants (Triathlon CR, Stryker) were used. The cohort included 89 patients (104 knees), with 21 men and 83 women. The mean age at surgery was 67 years (range 33–85), and the mean follow-up was 11.3 years (range 10–13). The study reported implant survivorship of 99.0% at a mean follow-up of 11.3 years, with only one early revision (1.0%) due to femoral component malrotation causing instability and recurrent hemarthroses, which was successfully treated by femoral component revision. One patient sustained a femoral periprosthetic fracture at 5 years, treated with open reduction and internal fixation without implant revision. Furthermore, Vermue et al.^32^ employed a retrospective cohort study including 143 eligible patients who underwent primary rKA-TKA with a rotating-platform deep-dish design. Patient demographics included a mean age of 71.3 ± 8.6 years, mean BMI of 30.0 ± 6.0 kg/m^2^ with 69% women and 31% men. Charnley classification (A- 40%, B1- 51%, C1- 1%, C3- 7%) and Devane classification (3- 36%, 4- 62%, 5- 2%) were reported. Follow-up lasted 5 ± 1 years, with 123 patients completing this. The study reported a revision-free survival rate at five years of 98%. Complications observed during the five-year follow-up included periprosthetic joint infection (1.6%), arthrofibrosis (1.6%), aseptic loosening of a cementless femoral component (0.8%) and patellar dislocation (0.8%). These results indicate excellent mid-term and long-term implant survivorship with a low rate of complications.

### 5.5 Robotic-assisted versus manual techniques

MA and rKA were performed using both traditional manual instrumentation (jig-based) and robotic-assisted techniques, each with distinct biomechanical implications, precision advantages and clinical trade-offs. Analysis of the included studies revealed a trend towards improved alignment accuracy and soft tissue balance with robotic-assisted techniques for both MA and rKA.^17^^,^^22^^,^^23^ However, the clinical significance of these refinements remains debated. For MA, robotic-assisted surgery consistently improved HKA accuracy, reducing outliers beyond ±3° of neutral alignment. However, there was no clear functional advantage over manual MA in PROMs. For rKA, robotic guidance allowed for more precise restoration of native knee morphology, particularly in tibial slope and femoral component rotation.^22^ While robotic-assisted rKA appeared to offer greater intraoperative control over bone resection and GB, its functional superiority over manual rKA remains unproved. Given the inherently patient-specific nature of rKA, the argument can be made that precision execution is more critical in rKA than MA thereby justifying the added cost and complexity of robotic systems. Deckey et al.^33^ deduced that robotic-assisted TKA achieved high precision in implant placement, with minimal deviations in coronal and rotational alignment. Furthermore, a meta-analysis conducted by Alrajeb et al.^34^ concluded that robotic TKA resulted in significantly better post-operative anatomical (OR − 0.82; 95% CI, −1.027 to − 0.58, p < 0.00001) and mechanical restoration (OR − 0.95; 95% CI, −1.49 to − 0.41, p < 0.0006) compared to conventional jig-based TKA. Additionally, knee range of motion (OR − 2.23; 95% CI − 4.89–0.43, p = 0.1) and femoral prosthesis position (OR − 0.98; 95% CI, −2.03–0.08, p = 0.07) also favoured robotic knees, though these differences did not reach statistical significance.

### 5.6 Future directions

To establish rKA as a widely accepted alignment philosophy, future research should prioritise RCTs with standardised surgical protocols to eliminate variability in execution, longitudinal studies (>10 years) evaluating polyethylene wear patterns, aseptic loosening rates and implant failure mechanisms, and standardised reporting of adverse events and effect sizes to facilitate comparisons across future studies.^35^^,^^36^ Additionally, patient selection criteria must be refined. Whilst rKA appeared to be beneficial for most primary TKAs, its role in high-demand patients, post-traumatic knees, and severe varus/valgus deformities is unclear.

## 6 Conclusion

The findings suggest that rKA achieved comparable or superior functional outcomes, particularly in terms of patient-reported satisfaction, early proprioception and knee awareness.^16^^,^^17^^,^^19^^,^^20^ Additionally, radiographic analysis confirmed that rKA remained within safe coronal and sagittal alignment tolerances, mitigating concern about excessive varus or valgus positioning.^34^ Importantly, there was no significant difference in short-term complication rates or implant survivorship between rKA and MA, although long-term durability remains uncertain 15, 19, 20, 22, 24.

Until further evidence clarifies its impact on implant longevity, rKA should be considered an evolving surgical technique rather than an absolute replacement for MA. Future research should focus on standardising surgical protocols, optimising patient selection, and integrating emerging technologies to enhance outcome predictability.

## Patient consent statement

N/A.

## Permission to reproduce material from other sources

N/A.

## Ethics statement

Due to the nature of the research, no ethics approval was needed.

## Clinical trial registration

Not applicable.

## Data availability statement

Not applicable; all relevant data are included in the manuscript.

## Credit author statement

Amaan Merchant: Conceptualisation, methodology, Writing - Original Draft, Writing - Review & Editing, formal analysis.

Maryam Imran: Conceptualisation, methodology, Writing - Original Draft, Writing - Review & Editing, Visualisation.

Mihika Joshi: Writing - Review & Editing.

Farhad Iranpour: Supervision.

Padmanabhan Subramanian: Supervision, Project administration.

## Funding statement

No funding was received for conducting this study.

## Conflict of interest disclosure

The authors have no relevant financial or non-financial interests to disclose.
